# Pervasive read-through transcription of *T-DNAs* is frequent in tobacco BY-2 cells and can effectively induce silencing

**DOI:** 10.1186/s12870-018-1482-3

**Published:** 2018-10-22

**Authors:** Vojtěch Čermák, Lukáš Fischer

**Affiliations:** Department of Experimental Plant Biology, Charles University, Faculty of Science, Viničná 5, 128 44 Prague 2, Czech Republic

**Keywords:** GFP, Inverted repeat, Promoterless, RNAi, Read-through transcription, *T-DNA*, Tobacco BY-2 cell line

## Abstract

**Background:**

Plant transformation via *Agrobacterium tumefaciens* is characterized by integration of commonly low number of *T-DNAs* at random positions in the genome. When integrated into an active gene region, promoterless reporter genes placed near the *T-DNA* border sequence are frequently transcribed and even translated to reporter proteins, which is the principle of promoter- and gene-trap lines.

**Results:**

Here we show that even internal promotorless regions of *T-DNAs* are often transcribed. Such spontaneous transcription was observed in the majority of independently transformed tobacco BY-2 lines (over 65%) and it could effectively induce silencing if an inverted repeat was present within the *T-DNA*. We documented that the transcription often occurred in both directions. It was not directly connected with any regulatory elements present within the *T-DNAs* and at least some of the transcripts were initiated outside of the *T-DNA*. The likeliness of this read-through transcription seemed to increase in lines with higher *T-DNA* copy number. Splicing and presence of a polyA tail in the transcripts indicated involvement of Pol II, but surprisingly, the transcription was able to run across two transcription terminators present within the *T-DNA*. Such pervasive transcription was observed with three different *T-DNAs* in BY-2 cells and with lower frequency was also detected in *Arabidopsis thaliana*.

**Conclusions:**

Our results demonstrate unexpected pervasive read-through transcription of *T-DNAs*. We hypothesize that it was connected with a specific chromatin state of newly integrated DNA, possibly affected by the adjacent genomic region. Although this phenomenon can be easily overlooked, it can have significant consequences when working with highly sensitive systems like RNAi induction using an inverted repeat construct, so it should be generally considered when interpreting results obtained with the transgenic technology.

**Electronic supplementary material:**

The online version of this article (10.1186/s12870-018-1482-3) contains supplementary material, which is available to authorized users.

## Background

*Agrobacterium tumefaciens* mediated transformation is a common method used to obtain transgenic plants. *Agrobacterium* transfers its *T-DNA* into a plant cell, where it can be integrated inside plant genome predominantly through double-strand break repair pathway (reviewed in [1]). Generally *T-DNAs* are stably introduced only in a small proportion of cells cocultivated with *Agrobacterium*. The *T-DNAs* are integrated at random positions in their genome [2, 3]. Subsequent regeneration of transgenic lines requires incorporation of a selection step to filter out untransformed cells/plants. This selection is usually achieved by incorporating antibiotic or herbicide resistance gene into the *T-DNA*. The requirement for the selection gene to be actively expressed then imposes bias on the selected transformants. *T-DNAs* of such transformants are preferentially present in regions with active transcription, especially near promoters and in regions with low nucleosome density [3, 4]. This probably leads to the unusually high success rate of various promoter- and gene-trap lines [5–7]. Although the transformants generated by *Agrobacterium* have lower number of insertions compared to other transformation methods, there are still many transformants with multiple insertions. Commonly the number of insertions per line varies between 1.4 and 4.9 [4, 8]. In case of multiple insertions, it is quite common for *T-DNAs* to integrate in a form of direct or inverted repeats in one position in the genome. Due to the way how *T-DNAs* are integrated, the most common form is the head-to-head (RB-to-RB) inverted repeat arrangement [9, 10]. Convergent read-through transcription of *T-DNAs* integrated as inverted repeats can induce silencing of homologous sequences via RNA interference (RNAi) [11, 12].

RNAi is an important mechanism in the regulation of gene expression in eukaryotic cells. In functional genomics, it is often used as a tool to modify expression of studied genes. The key players in RNAi are small RNAs (sRNAs), which can be formed by multiple pathways in plants, making the plant RNAi a very complex process (reviewed in [13]). Generally, a double stranded RNA (dsRNA) is needed to induce sRNA production in plants. There are many different ways to achieve dsRNA formation in a cell, the most efficient one is intermolecular pairing of transcripts coming from an inverted repeat [14].

Triggering RNAi by introduced “silencer constructs” can be used to knock out genes of interest or to study the mechanisms of RNAi itself. The large majority of RNAi studies were based on the model plant *Arabidopsis thaliana* that offers high quality genomic data and plenty of mutant lines, which are easily accessible to the research community. Few years ago, we started to test an alternative model, tobacco BY-2 cell line that has been successfully used in numerous studies focused on cellular processes [15]. The BY-2 cell culture is composed of relatively homogeneous mitotically proliferating cells [15, 16]. The absence of the gametophytic phase also prevents some types of epigenetic changes connected with this developmental stage [17–20]. The BY-2 cell line also allows simple analyses at the level of individual cells and assessment of a large number of independent transgenic lines (in the callus form) that can be easily generated, managed and analyzed [16]. Since the behavior of individual transgenic lines of any model is affected by the *T-DNA* copy number and the chromosomal environment of the insertion [21–23], analysis of a high number of independent transformed lines, which provides a more generalized picture, is recommended. Other advantages include easy and reliable analyses of fluorescence levels in these cells as well as simple ways to treat these cells with various chemicals. In situations, where the study is focused on analyses of general molecular and cellular mechanisms, the absence of the whole plant context may not have substantial impact on the appropriate generalization of the results.

The observation of pervasive read-through transcription of *T-DNAs* that we describe in this study, was discovered during our RNAi project focused on comparison of silencing potential of different silencing inducers. We supertransformed a BY-2 line stably expressing the *GFP* gene [24] with various silencers that were not controlled by a constitutive promoter as usual, but they were based on the XVE inducible system [25, 26], providing the possibility of highly reliable induction of RNAi by β-estradiol. The *GFP* reporter gene was used to allow simple visualization of silencing.

We generated hundreds of independent transgenic BY-2 lines (calli) with a goal to assess population responses to the induced expression of each silencing inducer (these data are not presented in this study). Surprisingly, we observed significant differences between the silencers already prior to their activation with β-estradiol with high frequency of silencing occurring in the calli carrying the inverted repeat construct. We found that this silencing correlated with spontaneous transcription of the silencer and here we show detailed analysis of this phenomenon.

## Methods

### Plasmid construction

**Fig. 1:**
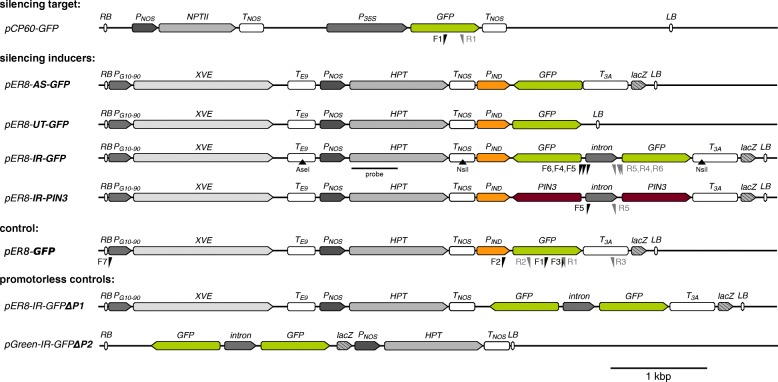
Schematic representation of *T-DNAs* used in this study. Functional elements, positions of primers used for reverse transcription and qPCR, position of a probe and restriction enzyme sites used for Southern hybridization are shown above and below the appropriate sequences. *pCP60-GFP*: construct for expression of *GFP* under the constitutive promoter. *pER8*: a set of *T-DNAs* used for the inducible expression: *pER8*-*AS-GFP*: sequence of *GFP* in antisense orientation; *pER8*-*UT-GFP*: sequence of *GFP* without terminator; *pER8*-*IR-GFP*: sequence of *GFP* arranged as inverted repeat; *pER8*-*IR-PIN3*: sequence of *PIN3* gene arranged as inverted repeat; *pER8*-*GFP*: sense *GFP*. The *pER8*-*IR-GFPΔP1* and *pGreen*-*IR-GFPΔP2* are the two promoterless controls with *GFP* sequence arranged as inverted repeat. *RB*: right border, *LB*: left border, *P*_*NOS*_: nopaline synthase promoter, *P*_*35S*_: *35S* promoter, *P*_*G10–90*_: synthetic constitutive promoter; *P*_*IND*_: inducible promoter activated by β-estradiol; *T*_*NOS*_: nopaline synthase terminator; *T*_*E9*_: *rbcS E9* terminator; *T*_*3A*_: *rbcs S 3A* terminator; *XVE*: estrogen receptor and transcriptional activator; *NPTII*: kanamycin resistance gene; *HPT*: hygromycin resistance gene; *GFP*: green fluorescent protein coding sequence; *intron*: intron from *Solanum tuberosum PsbO* gene; *LacZ*: fragment of bacterial β–galactosidase gene. All *T-DNAs* are at the same scale as indicated with 1kbp scale bar

All silencer *T-DNAs* (Fig. 1) were prepared similarly. First the full length *GFP* sequence was PCR amplified from *psmRS-GFP* plasmid [27] using primers with appropriate adapters (Additional file 1). The sequence was then inserted into the *pDrive* vector (part of QIAGEN PCR Cloning Kit) and subsequently transferred into the destination binary vector – either *pER8* [26] or *pGreen* [28] using the appropriate restriction endonucleases. The *AS-GFP* was cloned between *Xho*I and *Bcu*I in *pER8*, the *UT-GFP* was cloned between *Xho*I and *Pvu*II in *pER8*, the *GFP* was cloned between *Xho*I and *Bcu*I in *pER8* (as *Sal*I and *Nhe*I fragment derived from *pDrive IR-GFP*). The *IR-GFP* construct was first assembled in *pDrive*: the intron from *Solanum tuberosum PsbO* gene (cDNA GeneBank no. X17578.1) including approximately 20 bp from exons on both sides was AT-cloned into *pDrive* (in *Sac*I-*Kpn*I orientation). The intron sequence placed inside the inverted repeats was demonstrated to enhance the efficiency of silencing likely via facilitating dsRNA formation [29]. *IR1-GFP* was inserted first between *Xho*I and *Sal*I, then *IR2-GFP* was inserted between *Pst*I and *Bam*HI; the whole *IR-GFP* construct was then cloned between *Xho*I and *Bcu*I in *pER8*. The *IR-GFP-ΔP1* control was created by cloning the *IR-GFP* construct between *Sal*I and *Bcu*I in *pER8* (*Sal*I cleaves at the beginning of the inducible promoter). The *IR-GFP-ΔP2* control was created by cloning the *IR-GFP* construct in *pGreen 0129* between *Sac*I and *Kpn*I. All cloning experiments were completed using enzymes from Fermentas (Thermo Fisher Scientific) and *Escherichia coli* strain JM109. The resulting *T-DNAs* were confirmed by restriction and sequencing (Fig. 1).

### Plant material

Tobacco cell line BY-2 (*Nicotiana tabacum* L. cv. Bright Yellow 2) [15] was obtained from Prof. Zdeněk Opatrný, who had cultivated the line for more than 20 years. BY-2 calli were cultivated on agar plates (0.8 *w*/*v* agar; 6 cm diameter plates) with modified MS medium [30]. The calli were subcultured monthly. Suspension cell cultures were subcultured every seventh day (1 ml of cells into 30 ml of liquid media). The cultures were kept in darkness at 26 °C; suspensions were placed on the orbital shaker IKA KS501 at 110 rpm (IKA Labortechnik, Staufen, Germany; orbital diameter 30 mm).

Transformations of BY-2 suspension cells were carried out as described previously [24] using *Agrobacterium tumefaciens* strain C58C1 carrying a helper plasmid *pGV2260* [31] and appropriate binary vector (see above). After cocultivation with agrobacterium, the cells were plated on solidified medium containing 25 μg/ml hygromycin and 100 μg/ml cefotaxim and cultured for 3 weeks. Using this procedure, individual transformed cells form isolated macroscopic cell clusters (commonly called calli) that can be mostly regarded as genetically homogeneous clones [24].

The promoter from the *pER8* plasmid was induced by cultivating the calli on media with addition of 2 μM β-estradiol (from Sigma-Aldrich Cat. No. E2758). The β-estradiol was stored as 20 mM solution in DMSO, therefore, a corresponding amount of DMSO was added to the cultivation medium of the controls.

*Arabidopsis thaliana* (Col-0) plants were grown in Jiffy soil pellets under long-day conditions with illumination of 100 μm m^− 2^ s^− 1^ photosynthetically active radiation (OSRAM L 58 W/930).

### Measurement and analysis of fluorescence level

The BY-2 calli grown after transformation (see above) were transferred to new plates with 100 μg/ml cefotaxim (20 calli per plate) and cultivated for approximately 8 days. 200 calli (each representing different transgenic event) were used for each of the variants. Each plate was photo-documented separately using G:BOX (SynGene, Cambridge, UK) with blue excitation light (LED diodes with maximum at λ = 465 nm) and green emission filter (FILT525/GX; 510–540 nm). The images were processed using software NIS-Elements 3.10 (Laboratory Imaging, Prague, CZ). The average light intensity was measured for all the pixels from each callus. These data were statistically analyzed using R 3.1.2 and Pearson’s chi-square test. The threshold for fluorescent and non-florescent calli was set as the highest fluorescence intensity measured for wild-type BY-2 callus (these were used as controls cultivated alongside the transgenic calli).

### Transcription analysis

RNA was isolated from 100 mg of 10 to 18 days old calli (in the same experiment the calli were always in the same age) or from 50 mg of siliques of 40 days old *Arabidopsis thaliana* plants (younger siliques were selected – less than 10 days old, to ensure high proportion of dividing cells in the tissue) using NucleoSpin® RNA Plant kit (MACHEREY-NAGEL, Düren, DE). The procedure was performed according to manufacturer’s instructions including the on-column DNA digestion. The RNA was measured on NanoDrop 2000 (Thermo Fisher Scientific) to assess the concentration of the samples and to exclude contamination of the RNA by impurities. The integrity of the RNA was checked by gel electrophoresis using the “bleach gel” method [32]. For cDNA preparation, 1 μg of the total RNA was again treated with DNase I and half of the reaction mixture was then used as template for RevertAid Reverse Transcriptase (Fermentas, Thermo Fisher Scientific). The other half was exposed to the same treatment, but without adding the Reverse Transcriptase – this served as a control to check for DNA contamination. The final cDNA was diluted into the volume of 50 μl. Either oligo(dT) or specific primers were used for the cDNA synthesis. Some of the specific primers were designed to allow for distinction between sense and antisense transcripts and spliced and unspliced molecules (Additional file 1).

The quantification itself was done by qPCR, using LightCycler 480 (Roche) and iQ™ SYBR® Green Supermix (BioRad, Hercules, CA, USA). All the experiments were done while keeping the general qPCR guidelines in mind [33]. Reactions were completed in 10 μl volume, using 1 μl of cDNA as a template; all reactions were done in triplicate. The specificity of the PCR was verified by melting curve analysis (using the LightCycler 480 software) and also by checking randomly selected samples using gel electrophoresis. For each set of primers, there was appropriate negative control (WT and/or dH_2_O). The PCR efficiency for each amplicon and the Cq values for each sample were calculated using the software LinRegPCR 2015.3 [34]. The values for triplicates were averaged after correction for PCR efficiency. Samples in the triplicate with no amplification or only unspecific products were counted as zeroes; the samples with the majority of unspecific product was treated as one order of magnitude smaller (unspecific products appeared only for samples with high Cq values, over 30). Calculated concentrations were normalized to the expression of *NtEF1α*, so all the presented values show the relative level of given transcript to the level of *NtEF1α*. Results were then statistically compared using R 3.1.2 and Welch’s t-test. Positions of primers used for qPCR are indicated in Fig. 1 and their sequences listed in Additional file 1. Some primer sequences were taken over from previous studies [35, 36].

The BY-2 calli for transcriptional analysis were selected randomly from groups of silenced and non-silenced calli based on the presence or absence of GFP fluorescence. In case of transformants carrying sense *GFP* (*GFP* sequence in *pER8* XVE inducible system), the BY-2 calli and *Arabidopsis* lines were randomly selected from those that were able to induce *GFP* expression when grown on the induction medium supplemented with β-estradiol.

### Southern blot analysis

The Southern blot hybridization was done as described previously [37] with the following modifications: The DNA was isolated from 150 mg (FW) of BY-2 calli. 20 μg of genomic DNA per sample was separately digested by enzymes *Nsi*I and *Ase*I (New England Biolabs). The probe was prepared as a fragment of HPT gene using PCR with primers *HPT_probe_F* and *HPT_probe_R* (Additional file 1).

The Southern blot was interpreted as follows: tandem *T-DNA* inserted as direct repeat should give 6.8 kbp fragment with both *Nsi*I and *Ase*I, plus one fragment of unknown size for *Nsi*I and *Ase*I; head-to-head inverted repeat should give 7.3 kbp fragment when digested with *Nsi*I and two fragments of unknown size when digested with *Ase*I; tail-to-tail inverted repeat should give 9.6 kbp fragment when digested with *Ase*I and two fragments of unknown size when digested with *Nsi*I.

## Results

### Fluorescence in calli transformed with various *GFP* silencer constructs

To study various aspects of RNAi, we prepared three different silencer *T-DNAs* based on the XVE inducible system [26]. Specifically, the silencing should have been achieved through production of i) antisense RNA (*AS-GFP*), ii) non-polyadenylated sense RNA (*GFP* without any terminator; *UT-GFP*) and iii) hairpin RNA (inverted repeat with an intron separating the antisense and sense *GFP* fragment; *IR-GFP*). These *T-DNAs* were expected to trigger posttranscriptional silencing of the reporter *GFP* gene only under induction with β-estradiol. As a control, we also prepared a construct with inducible *GFP* in sense orientation and ended with terminator (*GFP*; Fig. 1).

**Fig. 2:**
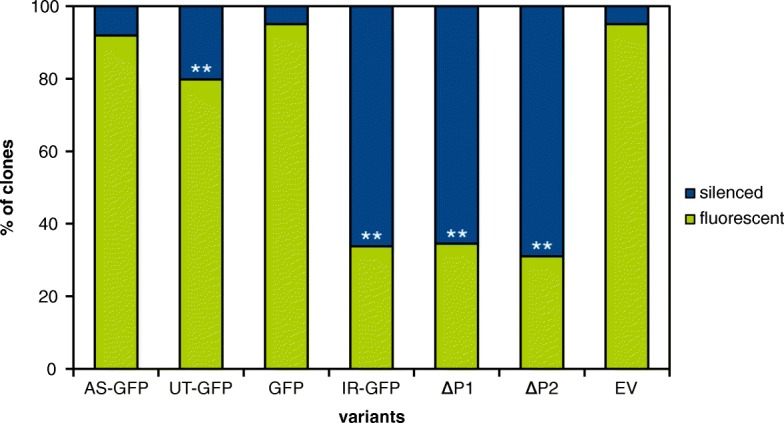
Frequency of spontaneous silencing of *GFP* in supertransformed BY-2 calli. Silencer and control *T-DNAs* (as described in Fig. 1) were supertransformed to BY-2 cell line stably expressing *GFP*. The fluorescence was assessed in independently transformed calli grown on non-inductive medium six weeks after transformation. The bars represent the percentage of fluorescent non-silenced calli (the lower part of the bar) and silenced calli (the upper part of the bar). The variants AS-GFP and UT-GFP represent averages of three biological replicas and the variants GFP and IR-GFP represent averages of four biological replicas (independent transformations). Each replica had 200 calli (with exception of EV with 160 calli). Variants that significantly differed from the EV control are marked with ** (*p* < 10^− 6^). *ΔP1: pER8*-*IR-GFPΔP1*; *ΔP2: pGreen*-*IR-GFPΔP2;* EV: empty vector *pER8*

A selected BY-2 cell line that has been stably expressing *GFP* (driven by *35S* promoter) for more than 8 years [24] was separately supertransformed with each *T-DNA.* We then analyzed GFP fluorescence in individual calli grown after the transformation - each representing independent transformation event (see Methods for details). On the control medium, where the calli were not exposed to β-estradiol, we expected similar fluorescence in all populations (hundreds of calli) carrying various silencers. However, we observed that *IR-GFP* population had strikingly lower frequency of calli with detectable GFP fluorescence compared to the other silencer and control *T-DNAs* (Fig. 2). All the differences between frequencies of GFP-positive calli in the IR-GFP population and those with other constructs were statistically significant (*p* < 10^− 40^). It should also be noted that some of the smaller differences between the variants were found significant as well: the UT-GFP variant compared to EV and GFP (*p* < 10^− 6^).

Since the calli grew on the medium without β-estradiol, the observed unexpectedly high proportion (over 65%) of spontaneously silenced IR-GFP calli prompted us to study this phenomenon further. Although XVE inducible system is considered to be reliable with very low leakiness of the inducible promoter [26], we had to exclude this possibility. We prepared two additional controls; i) we removed the inducible promoter from the *pER8* vector (*IR-GFPΔP1*) and ii) we cloned the promoterless *IR-GFP* into the empty *pGreen* vector (*IR-GFPΔP2*). After transformation to BY-2 cells, the number of spontaneously silenced independent calli was virtually the same as with the original *IR-GFP T-DNA* (Fig. 2, columns 5 and 6; the differences between the IR variants were not statistically significant), indicating that the silencing was independent of the presence of the inducible promoter and the *T-DNA* context.

To exclude the possibility that the transcription was initiated from elements that might be common for both *T-DNAs*, we compared their sequences. We found two homologous regions: i) the HPT expression cassette, which, however, differed between the two *T-DNAs* in its orientation relative to the *IR-GFP* sequence (Fig. 1) and ii) a short 156-nt fragment of bacterial β–galactosidase gene (*LacZ*) that was downstream of the *IR-GFP* in both *T-DNAs*. No promoter regulatory elements were predicted within this sequence by TSSP software (http://linux1.softberry.com).

### Transcription and splicing of *IR-GFP*

**Fig. 3:**
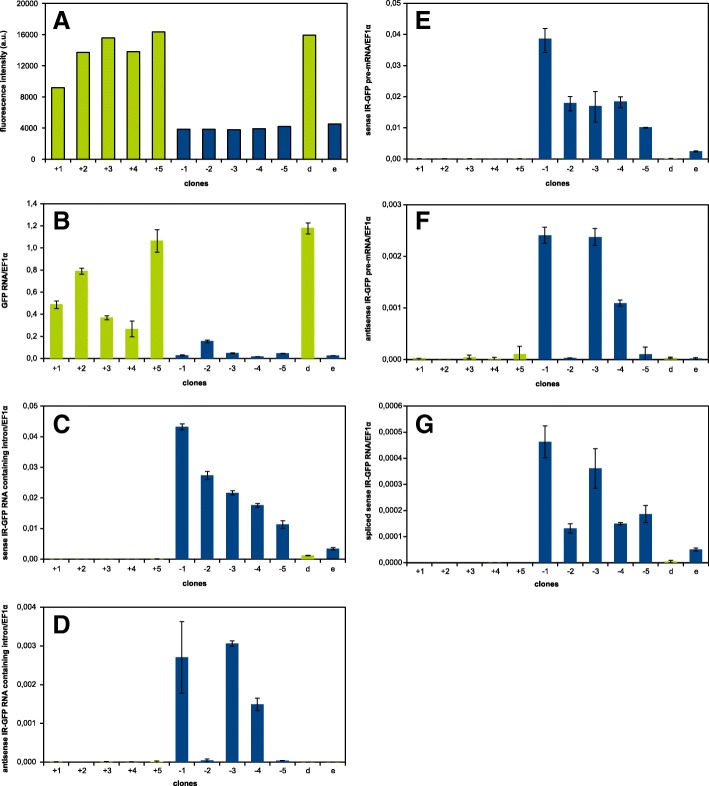
Analysis of selected BY-2 calli supertransformed with *pER8-IR-GFP*. RT-qPCR analysis of *IR-GFP* transcript levels in five randomly selected spontaneously silenced calli (marked from − 1 to − 5) and five non-silenced calli (marked from + 1 to + 5) and one additional non-silenced callus grown on the induction medium with β-estradiol (marked as “e”) and on the control medium with DMSO (marked as “d”). Each callus represented independent transformation event. **a** Fluorescence of analyzed calli (in arbitrary units), signals below 4000 represent the background; **b** the level of the sense *GFP* transcript (RT: oligo dT primer, qPCR: F1 and R1 primers); **c** the level of transcripts with the sense intron of *IR-GFP* (representing both the unspliced transcript and the spliced intron; RT: R5 primer, qPCR: F5 and R5 primers); **d** the level of transcripts with the antisense intron of *IR-GFP* (RT: F5 primer, qPCR: F5 and R5 primers); **e** the level of the sense *IR-GFP* unspliced transcript (RT: R5 primer, qPCR: F4 and R5 primers); **f** the level of the antisense *IR-GFP* unspliced transcript (RT: F5 primer, qPCR: F5 and R4 primers); **g** the level of the sense spliced transcript (RT: R6 primer, qPCR: F6 and R6 primers)

Transcription analysis was done in five non-silenced and five silenced calli that were randomly selected from populations with and without detectable GFP fluorescence. The results showed that the levels of *GFP* transcripts roughly matched the GFP fluorescence intensities, with the lowest transcription being detected in the silenced calli (Fig. 3a and b). To see whether the silencing correlated with transcription of the *IR-GFP*, we generated cDNAs using primers specific for the *GFP* hairpin transcribed in both the sense and antisense orientation (the “sense” and “antisense” transcripts were relative to the intron separating the *GFP* sequences in the *IR-GFP*). Transcript levels were analyzed using qPCR (Fig. 3b-h) with primers designed to allow separate quantification of i) spliced transcripts, ii) unspliced transcripts and iii) intron-containing molecules (i.e. nascent transcripts and spliced introns; Fig. 1). The *IR-GFP* transcripts were detected in all the spontaneously silenced calli at levels even higher than in the callus, where the *IR-GFP* transcription was induced with β-estradiol. In contrast, almost undetectable levels (three to four orders of magnitude lower) were found in all non-silenced calli with detectable GFP fluorescence (Fig. 3a and c, for the comparison of averaged relative transcript levels and their statistical comparison see Additional file 2). Surprisingly, in three of the five silenced calli, the *IR-GFP* was clearly transcribed also in the “antisense” direction (from the terminator towards the promoter), although at lower levels than it was transcribed in the “sense” orientation. There was no impact of β-estradiol treatment on the hairpin transcription in the antisense orientation in the control line (Fig. 3d).

The results obtained from detecting the intron sequence were similar to those obtained from detecting the unspliced transcript (PCR product spanning the exon-intron boundary), this was also true for the antisense direction (Fig. 3e and f). Such results could indicate either fast degradation of the spliced intron or that the intron was not spliced at all. To see if the splicing actually took place, we ran qPCR over the supposed exon-exon boundary. Using the “sense” cDNA, we were indeed able to amplify the specific product corresponding to the spliced hairpin RNA in the silenced calli at similar levels in both the spontaneously silenced calli and in the estradiol-induced control. The ratio of spliced/unspliced transcripts was between 0.7 and 2.1% for the spontaneously transcribed lines and 2.1% for the estradiol-induced control. It suggested that at least some of the *IR-GFP* transcripts were spliced correctly in the spontaneously silenced calli (Fig. 3g; the specificity of the qPCR was verified, see Methods).

### Spontaneous transcription of *IR-PIN3* and sense *GFP*

**Fig. 4:**
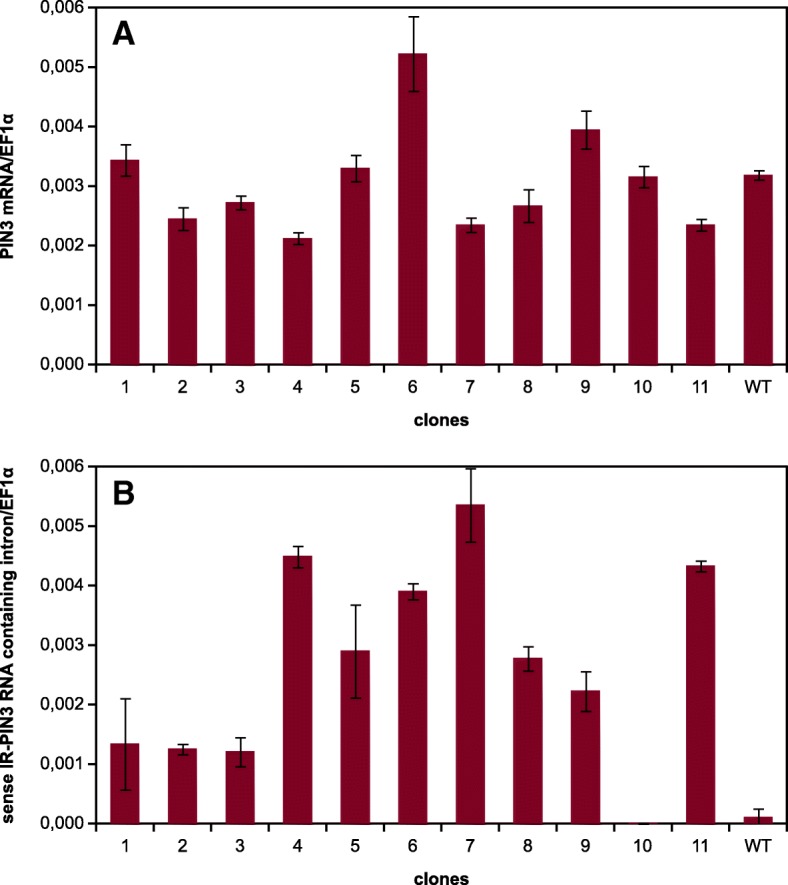
Transcription analysis of selected BY-2 calli transformed with *pER8-IR-PIN3.* RT-qPCR analysis of transcript levels in eleven randomly selected calli and an untransformed BY-2 callus as a control. **a** The level of the *PIN3* mRNA (RT: oligo dT primer, qPCR: PIN3_F and PIN3_R primers); **b** the level of the sense intron of the IR-PIN3 (representing both the unspliced transcript and the spliced intron; RT: R5 primer, qPCR: F5 and R5 primers)

The spontaneous transcription and subsequent silencing could be specific for our *IR-GFP* construct or it could represent more general phenomenon. Therefore, we tested the occurrence of spontaneous transcription of another inverted repeat construct, *IR-PIN3* prepared from a fragment of tobacco *PIN3* gene placed in the *pER8* plasmid (*NtPIN3bT*; kindly provided by Jan Petrášek) that we used to transform wild-type BY-2 cells. We analyzed the expression of the native *PIN3* gene and the *PIN3* hairpin in 11 randomly selected independent calli using RT-qPCR. We observed expression of the *PIN3* hairpin in the majority of analyzed calli. The relative transcription levels seemed to be somewhat lower (from 2 to 20 times lower) compared to the *GFP* hairpin in the *IR-GFP* silenced calli (Fig. 4a-c, for the comparison of averaged relative transcript levels see Additional file 2). However, the comparison was based on the amplification of the intron sequence, so the real transcription levels of the two hairpins could differ due to various efficacy of their splicing. The *IR-PIN3* transcription did not cause strong silencing of *PIN3* in contrast to the situation with *IR-GFP* and *GFP*. Only a weak decrease in *PIN3* expression could be observed in some calli with the highest level of the PIN3 hairpin (see Fig. 4a and b). However, the averaged *IR-PIN3* transcript levels did not significantly differ between calli with higher and lower *PIN3* transcription (Additional file 2).

**Fig. 5:**
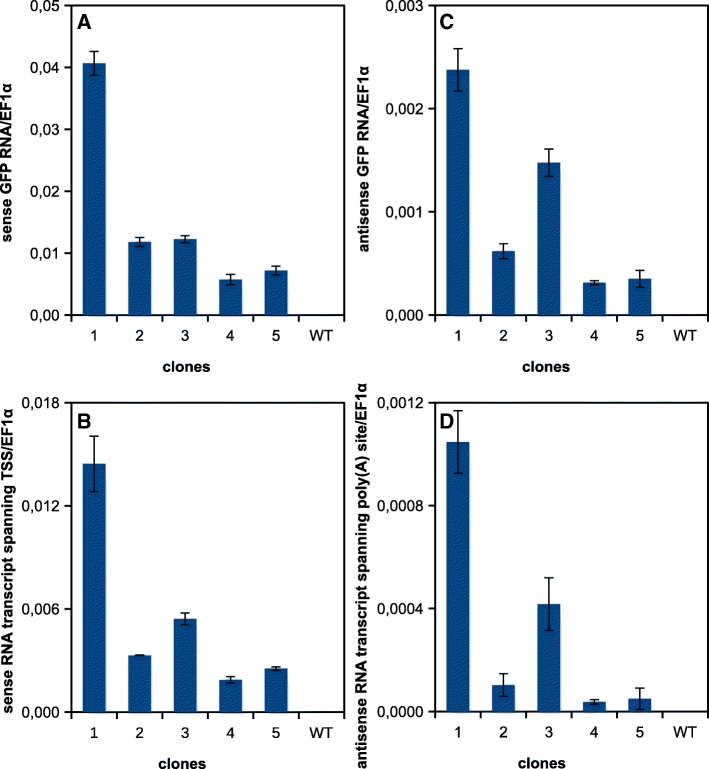
Transcription of *GFP* gene in selected BY-2 calli transformed with *pER8-GFP.* RT-qPCR analysis of transcript levels in five selected independent calli grown on the medium without β-estradiol. **a** The level of the sense *GFP* transcript (RT: R1 primer, qPCR: F1 and R1 primers); **b** the level of the sense transcript containing the region 50 nt upstream of transcription start site (*TSS*) of the inducible promoter (RT: R1 primer, qPCR: F2 and R2 primers); **c** the level of the antisense *GFP* transcript (RT: F1 primer, qPCR: F1 and R1 primers); **d** the level of the antisense transcript containing the region 50 nt downstream of the last poly(A) signal of T3A terminator (RT: F1 primer, qPCR: F3 and R3 primers)

To assess if the observed high frequency of spontaneous transcription was specifically connected with inverted repeat arrangement of introduced transgenes or if it was more general phenomenon in our experimental system, we analyzed *T-DNA* transcription in lines carrying sense *GFP* in *pER8* XVE inducible system (Fig. 1). After transformation into wild-type BY-2 cell line, there were no calli with detectable GFP fluorescence that would indicate spontaneous transcription connected with subsequent translation into functional GFP protein without β-estradiol treatment. However, RT-qPCR analysis showed that, at the transcriptional level, the sense *GFP* construct behaved similarly to the inverted repeats. The transcription was detected in all five calli and in both sense and antisense directions. The relative transcript levels were similar to those described for the hairpins (Fig. 3c-f, Fig. 5a and c, Additional file 2). This showed that the transcription was not connected with the inverted-repeat character of the sequence present in the *T-DNA*.

**Fig. 6:**
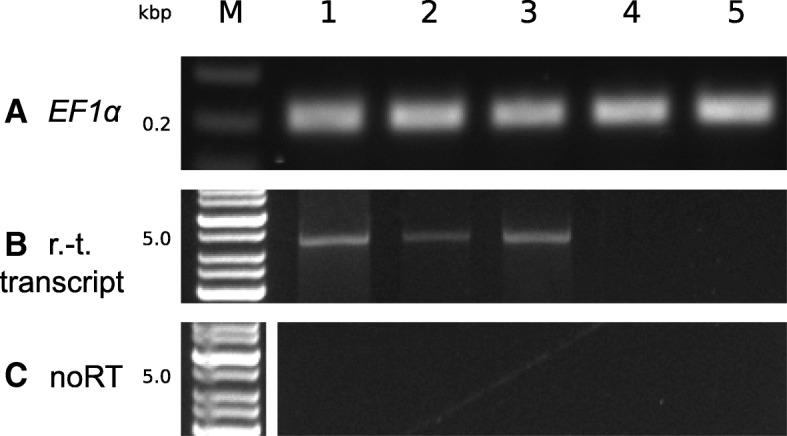
Read-throught transcripts of the *pER8 T-DNA* in selected BY-2 calli transformed with *pER8-GFP*. Semiquantitative RT-PCR analysis of transcript levels in five independent calli untreated with β-estradiol (the same calli as in Fig. 5). cDNA was prepared using R1 primer. **a** The level of the *EF1α* transcript (internal standard); **b** the level of read-through (r.-t.) *pER8 T-DNA* transcript (F7 and R2 primers); **c** amplification of RNA samples that were not treated with reverse transcriptase to ensure that there was no DNA contamination

To better understand the nature of the spontaneous transcription, we tried to identify the transcription start site by 5’RACE, but the attempts failed despite intensive optimization. Therefore, we investigated whether the transcript originated within or outside the transcription unit in *pER8 T-DNA*. For this purpose, we used a primer that matched to the region 50 nt upstream of the transcription start site (*TSS*) for the sense transcript and a primer matching to the *3A* terminator region 50 nt downstream of the last predicted poly(A) signal for the antisense transcript (as predicted by PASPA software: http://bmi.xmu.edu.cn/paspa/index.html; [38]). We detected transcripts from both regions and their levels in individual calli correlated with previously detected transcripts of the *GFP* gene. This suggested that the spontaneous transcription originated (at least partially) outside of the transcription unit in *pER8 T-DNA.* The nearest *ATG* is 99 bp upstream from the proper *ATG*, so transcripts originating from this 99-bp long region could be theoretically translated into the proper GFP protein, but we have never detected GFP fluorescence in such lines, which indicated *TSS* being more upstream. Thus, we tried to roughly localize the position of *TSS* within the *T-DNA*. We designed a set of forward primers along the *T-DNA* for amplification from *cDNA* prepared with a reverse primer specific to the *GFP* sequence. Surprisingly, we obtained products even with the most upstream primer located near the border sequence and preceding any promoter present in the *T-DNA* (Fig. 6). This PCR product was more than 4 kbp long and included two upstream transcription units for the *XVE* receptor and the *HPT* gene. Thus the transcription was able to overcome two transcription terminators (Fig. 1).

### Determination of *T-DNA* copy number and arrangement

To better understand potential reasons of this transcription, we estimated copy number and arrangement of *T-DNAs* by Southern hybridization in three silenced and three non-silenced lines (selected according to the data in Fig. 3). The results indicated that the read-through transcription and spontaneous silencing might be connected with higher *T-DNA* copy number (Additional file 3); 1 to 3 copies were detected in non-silenced lines and 3 to approximately 8 copies in silenced lines. Further analyzes of the size and number of hybridizing bands in individual lines suggested that *T-DNA* arrangements allowing read-through transcription from one *T-DNA* to another, i.e. direct *T-DNA* repeats and inverted tail-to-tail repeats, were not present in lines − 1 and + 2. In the other four lines the situation was not clear due to multiple insertions (− 3, − 4, + 3) or the presence of truncated copies (− 4, + 1), but theoretically direct repeat could be present in lines − 3 and − 4 and tail-to-tail repeat in line − 4 and + 3.

### Spontaneous transcription in *Arabidopsis thaliana*

**Fig. 7:**
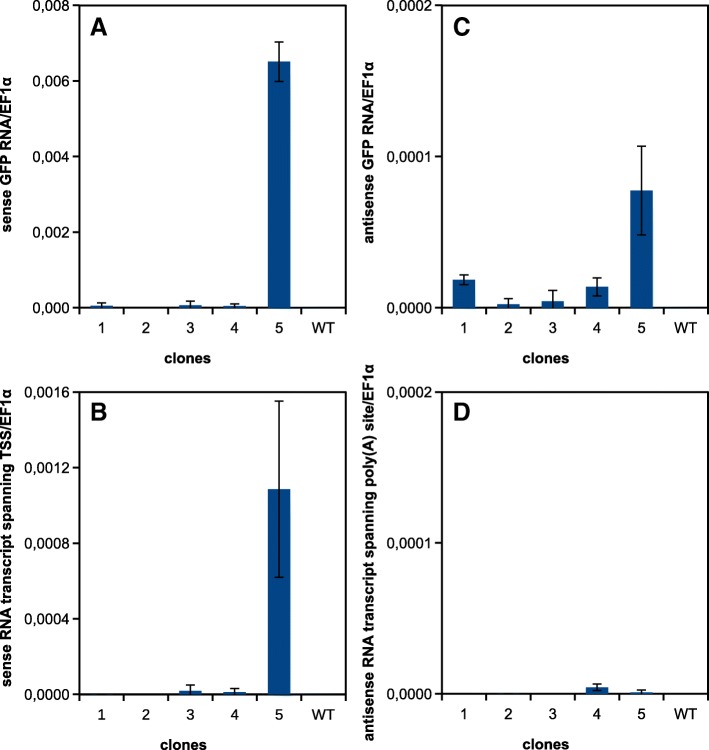
Transcription of *GFP* gene in siliques of selected *Arabidopsis thaliana* transformants with *pER8-GFP.* RT-qPCR analysis of transcript levels in five selected plants untreated with β-estradiol. **a** The level of the sense *GFP* transcript (RT: R1 primer, qPCR: F1 and R1 primers); **b** the level of the sense transcript containing the region 50 nt upstream of transcription start site (TSS) of the inducible promoter (RT: R1 primer, qPCR: F2 and R2 primers); **c** the level of the antisense *GFP* transcript (RT: F1 primer, qPCR: F1 and R1 primers); **d** the level of the antisense transcript containing the region 50 nt downstream of the last poly(A) signal of *T*_*3A*_ terminator (RT: F1 primer, qPCR: F3 and R3 primers)

To assess wider significance of our observation we analyzed the transcription in a different model organism – *Arabidopsis thaliana* plants carrying the same *T-DNA* with sense *GFP* in *pER8* XVE inducible system. We randomly selected five transformants as before. RNA was isolated from immature siliques of plants grown in soil without any exposure to β-estradiol. As in the experiment with BY-2 cell line, we analyzed the *GFP* transcription in both the sense and antisense directions and also the transcripts from the regions spanning the canonical transcription start site and the poly(A) signal. We were able to detect transcripts in the sense direction from both the *GFP* sequence and from the region spanning the *TSS* and in the antisense direction from the *GFP* region (Fig. 7a-c). However transcription was detected in only one transformed line at the level comparable to the BY-2 callus with the lowest transcript level. The transcripts in the antisense direction over the poly(A) signal were almost undetectable (Fig. 7d). Transcription in leaves was somewhat lower (Additional file 4) than in immature siliques, which have higher proportion of actively dividing cells (similarly to the BY-2 cell line).

## Discussion

### *GFP* silencing was connected with spontaneous transcription of *IR-GFP T-DNA*

This study was prompted by observation of massive *GFP* silencing occurring after the introduction of *IR-GFP* construct into BY-2 cells with stable expression of *GFP*. Such observation was surprising because the *IR-GFP* was controlled by *XVE* system that is considered to be one of the most reliable, i.e. the least suffering from leaky transcription [25, 26]. Although it is rare in the XVE system, there are reports showing that leaky expression can occur [39, 40]. To exclude this possibility, we employed additional controls; two *IR-GFP* constructs without the presence of the inducible (or any other) promoter. The results were identical in both cases (Fig. 2), clearly showing that the silencing did not occur as a result of the inducible promoter leakiness.

The observed silencing of the *GFP* clearly correlated with transcription of the *IR-GFP*, suggesting that silencing occurred as a result of this transcription at the posttranscriptional level (Fig. 3). By testing *PIN3* inverted repeat, we excluded the possibility that the *GFP* sequence itself was the cause of the spontaneous transcription. The *IR-PIN3* was also clearly transcribed in vast majority of analyzed calli, although we did not observe strong *PIN3* silencing as in the case of *GFP* (Fig. 4). Repeat regions are often targets of silencing [13, 41], so we asked whether the repetitive nature of our silencing inducers (*IR-GFP* and *IR-PIN3*) could be the reason for why they were spontaneously transcribed. But RT-qPCR analysis showed that single sense *GFP* gene was transcribed at similar frequency and level in BY-2 calli, so we assumed that neither the repeat structure of the sequence was necessary for the spontaneous transcription (Fig. 5). Since equal frequency of *GFP* silencing was observed with two different *T-DNAs* carrying the *IR-GFP*, we could conclude that the spontaneous transcription was unlikely connected with any regulatory elements present within the *T-DNAs.*

### Expression of endogenous *PIN3* was more resistant against silencing induced by spontaneous transcription of *IR-PIN3*

Unlike *IR-GFP*, spontaneous transcription of *IR-PIN3* resulted in only small decrease or unaffected level of *PIN3* mRNA (Fig. 4 and Additional file 2). The level of *IR-PIN3* transcription was somewhat lower than the transcription of *IR-GFP*, but the lowest transcript level of *IR-GFP* able to silence *GFP* expression was lower than the highest transcript level of *IR-PIN3* that was not able to silence *PIN3* expression (Figs. 3 and 4). The *IR-PIN3* transcript could be theoretically less efficient in forming dsRNA and producing siRNAs. However, after treatment with β-estradiol, the same construct worked as an effective inducer of silencing (Jan Petrášek, personal communication). An alternative explanation is that the difference was related to the higher sensitivity of artificially introduced *GFP* transgene to silencing. This could be connected with the absence of introns in the *GFP* gene. The presence of an intron and its splicing can suppress production of secondary siRNAs that might be necessary to amplify silencing when the transcript level of the inducer is low [42–44].

### Pol II was involved in the spontaneous transcription

Plant specific RNA Polymerases IV and V provide wide-spread transcription of genomic DNA important for de novo DNA methylation and maintenance of heterochromatin [45–47]. The transcripts we detected thus may originate from these polymerases. However, the size of the transcripts we detected exceeded the size typical for Pol IV and V transcripts, i.e. tens of nt long up to few hundreds nt respectively [48, 49]. Moreover, at least some transcripts in the sense direction were spliced (Fig. 3g), which is typical for Pol II transcription. The ratio between the spliced and unspliced transcripts was similar in calli with spontaneous transcription and in estradiol-induced control line, where transcription was fully done by Pol II (compare columns e in Fig. 3c and g). The very low levels of spliced transcripts likely resulted from instant processing by the DCLs [13]. Amplification of transcripts from cDNA samples prepared by reverse transcription with oligo dT primers further indicates that at least a portion of the transcripts were polyadenylated (Additional file 5). Although we cannot exclude the possibility that other polymerases contributed to the spontaneous transcription, Pol II surely participated, because splicing and the presence of a polyA tail are characteristic features for its transcription.

### Pervasive character of spontaneous transcription was possibly connected with specific chromatin state of some *T-DNAs*

The level of the spontaneous transcription strongly differed between independently transformed lines carrying the same *T-DNA*, indicating that the spontaneous transcription was not general, but rather specific to certain insertion events/loci.

Our attempts to find the transcription start site indicated that it was located outside of the *T-DNA*. It was previously demonstrated that transcripts originating from adjacent genomic regions could read-through across the border regions of *T-DNA* inserts and affect expression of transgenes present near the border sequence [50, 51]. Such transcription can also originate from a neighboring *T-DNA*. For this to occur in our system, the *T-DNAs* would have to be arranged either as tandem direct repeats or as tail-to-tail inverted repeats. But, it seems unlikely, that two thirds of transformed lines would contain such *T-DNA* arrangement and also our Southern hybridization did not indicate increased presence of such arrangements among the silenced lines. Therefore, we hypothesize that it was just the higher *T-DNA* copy number detected in silenced lines, which increased the probability that at least one *T-DNA* copy was inserted in a genomic region supporting the spontaneous transcription.

In the case of sense transcripts spanning the *pER8 T-DNA,* the polymerase was able to run across two terminators, yet the frequency of spontaneous transcription (silencing) was the same as in the case of the *pGreen T-DNA*, where *IR-GFP* was located near the border sequence. It should be also noted that the spontaneous transcriptional activity was often bidirectional, and that there was a correlation between the sense and antisense transcription levels in many calli. Based on these observations, we hypothesize that the spontaneous transcription was connected with specific chromatin state in some *T-DNA* insertion loci.

Our previous study indicated that the establishment of epigenetic marks can be accidental and at least in some cases independent of the chromosomal environment, since different epigenetic states could be established in the same insertion locus [24]. *T-DNAs,* when being inserted into the chromosome, are likely free of any epigenetic marks; as such, the formation of new marks may either reflect the chromatin state in the insertion locus or the new chromatin may be labelled with marks specific for new DNA insertions. This could make it prone to basal transcription by polymerase II that can somehow ignore terminators, thus forming long transcripts, irrespective of canonical transcription units present in the sequence. It is known, that facultative termination of long non-coding RNAs (lnRNAs) can be involved in regulation of gene expression and silencing [52–54] and that the chromatin state does have an effect on transcription termination [55]. More specifically, it was demonstrated that IBM2 allows Pol II to read-through silenced transposable elements inserted in introns of genes [56] and recent report showed that similar mechanism can also work for *T-DNAs* in introns of genes [57]. So we hypothesize that this long read-through transcription of newly integrated *T-DNAs* could be a component of genomic safety mechanisms evolved to allow silencing of newly integrated invasive DNAs (like transposable elements, TE). Such long polymerase II transcripts could be recognized by ubiquitous TEs-derived hc-siRNAs to initiate their silencing. Being an internal part of long transcript, there would be a little risk of translation of the TE transcript into executive proteins that would activate their replication/transposition.

### Read-through transcription of inserted *T-DNAs* might be a more general phenomenon

The important question is how widespread and how significant this phenomenon can be. We tested three types of gene constructs (*IR-GFP*, *IR-PIN3* and *GFP*) within two different *T-DNAs* (*pER8* and *pGreen*) using two different model organisms (BY-2 tobacco cell line and *Arabidopsis thaliana* plants). The results with various *T-DNAs* in BY-2 tobacco cell line were highly consistent, showing high frequency of read-through transcription and relatively high transcript levels. Additional analysis done with one *T-DNA* in *Arabidopsis thaliana* slightly differed, showing lower levels of transcription and lower occurrence among analyzed transformed plants. This could be connected with the physiological state of cells used for analyses (cell line vs. intact plant tissues) or different transcriptional regulation in species with dissimilar genome size (tobacco vs. *Arabidopsis thaliana*).

We also searched previous studies, which used inducible systems in plants to find further support for our observation. But most of the studies did not report the levels of RNA and if yes, then it is unclear if the transcript was not detected due to its absence or because it was below the detection limit. For example, Dohi et al. [23] did not detect any transcripts from the uninduced XVE system in BY-2 cell lines using Northern blot with DIG-labeled probes, but neither we were able to detect this transcription using the same detection method (data not shown), yet the RT-qPCR method clearly confirmed presence of these transcripts. In accord with our results, Kubo et al. [58] also detected low levels of transcripts from uninduced XVE system in *Physcomitrella patens* using RT-qPCR.

Additional strong support comes from early studies on silencing that used promoterless constructs as controls; they analyzed if silencing was caused by RNA or if it could be mediated directly by DNA-DNA interaction [11, 51, 59–62]; these works were done in *Petunia*, tobacco, *Arabidopsis thaliana* and *Neurospora*. Of the works mentioned above, only Cogoni et al. [51] partially characterized the origin of the transcripts causing the silencing in *Neurospora crassa*, by showing that they likely originated outside of the transgene. Whereas some studies did not observe silencing with promoterless constructs [63, 64], several other studies indeed reported induction of silencing by constructs or *T-DNAs* arranged as inverted repeats even without the presence of a promoter sequence [11, 59, 60]. All these old observations could be easily explained as read-through pervasive transcription of inverted repeats that produced hairpin RNAs.

Our data illustrate high incidence of pervasive read-through transcription of *T-DNAs* in BY-2 cells. These data are supported by very large numbers of analyzed independently transformed lines, 200 to 800 calli per each variant. In plants, only Sijen et al. [60] previously showed transcripts that likely originated from the silencer locus. As such, our report provides a considerable contribution to a more than fifteen years unsolved enigma, which has been experimentally overlooked with exception of some studies on DNAi [65]. We presume that the phenomenon of read-through transcription of *T-DNAs* is general, but specifically manifests only when working with *IR*, which has high potential to effectively induce silencing [14]. In contrast, low-level transcription of other constructs easily passes unnoticed, as the long-range character of transcripts disables translation into functional proteins that could visibly affect the phenotype.

## Conclusions

We observed unexpectedly high frequency of read-through low-level transcription of several different *T-DNAs* in tobacco BY-2 cell lines and to some extent also in *Arabidopsis thaliana*. We show that this transcription was at least partially catalyzed by Pol II, which was able to read-through two standard terminators. We speculate that this unusual transcription was connected with establishment of specific chromatin state in some *T-DNA* insertions*.* Such read-through transcription could be for example a component of a safety mechanism for recognition and silencing of invasive DNA insertions. In the case of *T-DNAs* containing *IR-GFP*, the spontaneous read-through transcription was sufficient to initiate very efficient silencing of the *GFP* gene (*in trans*) in the majority of analyzed BY-2 calli. From the practical view, it is important that the researchers using inducible silencing systems should be aware of this phenomenon as it can in some cases largely affect the obtained results.

## Additional files

Additional file 1: List of primers used in this study. (XLS 13 kb)

Additional file 2: Comparison of mean transcript levels from the analyzed T-DNAs. (A) Means of the IR transcript levels presented in Fig. 3 and Fig. 4. The means were calculated separately for the spontaneously silenced independent calli (i.e. 5 biological replicates, marked -) and non-silenced calli (marked +), one additional non-silenced callus grown on the induction medium with β-estradiol (marked as “e”) and on the control medium with DMSO (marked as “d”) is presented alongside. For the IR-PIN3, the category “spontaneously silenced calli” include half of the calli with the lowest expression of *PIN3* mRNA, the rest of the calli is part of the “non-silenced calli” category. Note that the higher expression of *IR-PIN3* in the category “spontaneously silenced calli” is not statistically significant. (B) Means of the transcript levels presented in Fig. 5. In all the experiments, intron from the inverted repeat (for both *IR-GFP* and *IR-PIN3*) is amplified with the same set of primers, so direct comparison of the transcript levels is possible. Also all the qPCR data are corrected for PCR efficiency (see Methods), so approximate comparison of quantities for different transcripts is also possible. The error bars represent standard deviations. (PDF 63 kb)

Additional file 3: Southern hybridization of total genomic DNA from BY-2 calli transformed with *pER8-IR-GFP.* Silenced independent calli marked with “-” and non-silenced calli marked with “+” (see Fig. 3). A DIG-labelled probe of the *HPT* gene was hybridized with DNA cleaved by *Nsi*I (N) and *Ase*I (A). *T-DNA* copy number was estimated as the number of hybridizing bands. The presence of repeats was analyzed as follows: tandem direct repeat should give 6.8 kbp fragment with both *Nsi*I and *Ase*I, plus one fragment of unknown size for *Nsi*I and *Ase*I; head-to-head inverted repeat should give 7.3 kbp fragment when digested with *Nsi*I and two fragments of unknown size when digested with *Ase*I; tail-to-tail inverted repeat should give 9.6 kbp fragment when digested with *Ase*I and two fragments of unknown sizes when digested with *Nsi*I. (PDF 341 kb)

Additional file 4: Transcription of *GFP* gene in leaves of selected *Arabidopsis thaliana* transformants with *pER8-GFP.* RT-qPCR analysis of transcript levels in five selected lines untreated with β-estradiol. (A) The level of the sense *GFP* transcript; (B) the level of the sense transcript containing the region 50 nt upstream of transcription start site (*TSS*) of the inducible promoter; (C) the level of the antisense *GFP* transcript; (D) the level of the antisense transcript containing the region 50 nt downstream of the last poly(A) signal of *T*_*3A*_ terminator. (PDF 39 kb)

Additional file 5: Detection on polyA-tailed *GFP* transcripts in selected BY-2 calli transformed with *pER8*-*GFP.* Semiquantitative RT-PCR analysis of transcript levels in five independent calli untreated with β-estradiol (the same calli as in Fig. 5). cDNA was prepared using oligo *dT* primers. (A) The level of the *Actin* transcript (internal standard); (B) the level of the *GFP* transcript; (C) amplification of RNA samples that were not treated with reverse transcriptase to ensure that there was no DNA contamination. (PDF 41 kb)

## Abbreviations

AS: antisense
IR: inverted repeat
LB: left border sequence of T-DNA
RB: right border sequence of T-DNA
UT: unterminated (without terminator)
