# Supplementary figures and images for: Pervasive read-through transcription of T-DNAs is frequent in tobacco BY-2 cells and can effectively induce silencing
Source: BMC Plant Biol. 2018 Oct 22;18:252. doi: 10.1186/s12870-018-1482-3 (PMC6196474; doi:10.1186/s12870-018-1482-3)

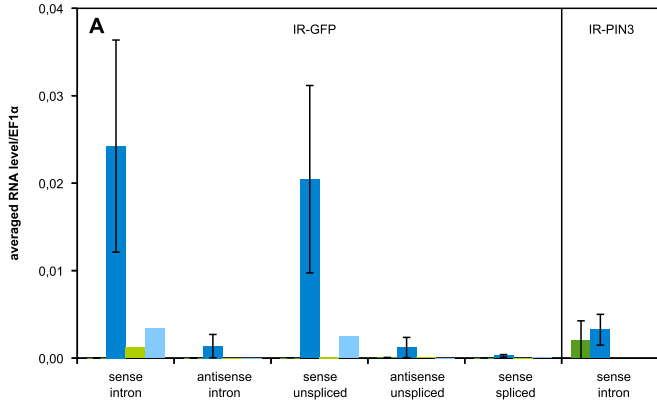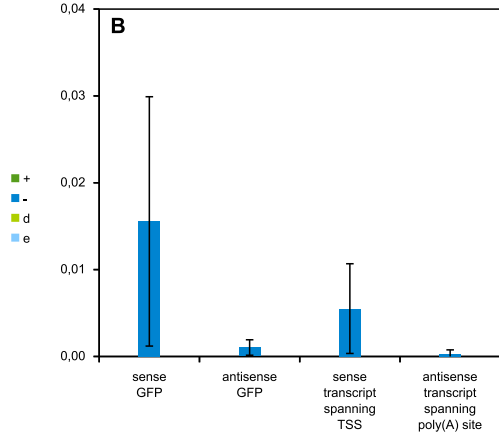

Supplement: Supplementary file 2 — Comparison of mean transcript levels from the analyzed T-DNAs. (A) Means of the IR transcript levels presented in Fig. 3 and Fig. 4. The means were calculated separately for the spontaneously silenced independent calli (i.e. 5 biological replicates, marked -) and non-silenced calli (marked +), one additional non-silenced callus grown on the induction medium with β-estradiol (marked as “e”) and on the control medium with DMSO (marked as “d”) is presented alongside. For the IR-PIN3, the category “spontaneously silenced calli” include half of the calli with the lowest expression of PIN3 mRNA, the rest of the calli is part of the “non-silenced calli” category. Note that the higher expression of IR-PIN3 in the category “spontaneously silenced calli” is not statistically significant. (B) Means of the transcript levels presented in Fig. 5. In all the experiments, intron from the inverted repeat (for both IR-GFP and IR-PIN3) is amplified with the same set of primers, so direct comparison of the transcript levels is possible. Also all the qPCR data are corrected for PCR efficiency (see Methods), so approximate comparison of quantities for different transcripts is also possible. The error bars represent standard deviations. (PDF 63 kb) [file 12870_2018_1482_MOESM2_ESM.pdf]

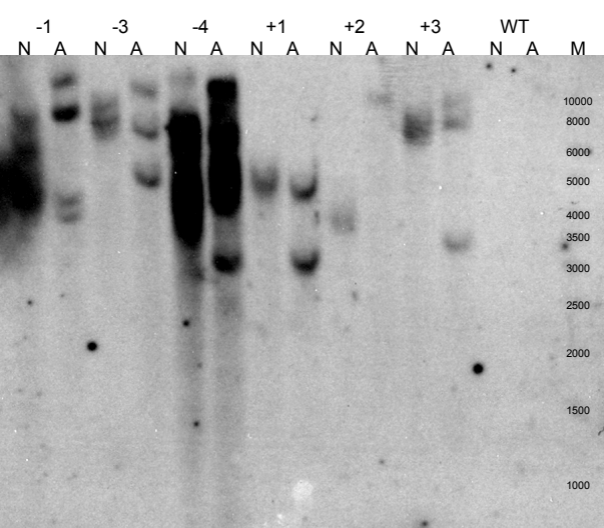

Supplement: Supplementary file 3 — Southern hybridization of total genomic DNA from BY-2 calli transformed with pER8-IR-GFP. Silenced independent calli marked with “-” and non-silenced calli marked with “+” (see Fig. 3). A DIG-labelled probe of the HPT gene was hybridized with DNA cleaved by NsiI (N) and AseI (A). T-DNA copy number was estimated as the number of hybridizing bands. The presence of repeats was analyzed as follows: tandem direct repeat should give 6.8 kbp fragment with both NsiI and AseI, plus one fragment of unknown size for NsiI and AseI; head-to-head inverted repeat should give 7.3 kbp fragment when digested with NsiI and two fragments of unknown size when digested with AseI; tail-to-tail inverted repeat should give 9.6 kbp fragment when digested with AseI and two fragments of unknown sizes when digested with NsiI. (PDF 341 kb) [file 12870_2018_1482_MOESM3_ESM.pdf]

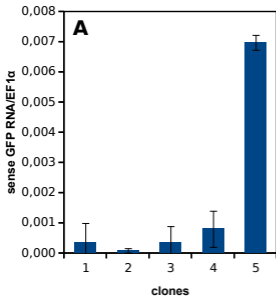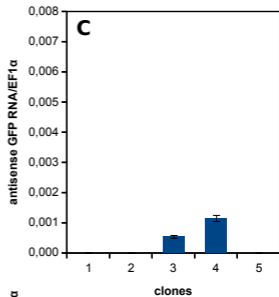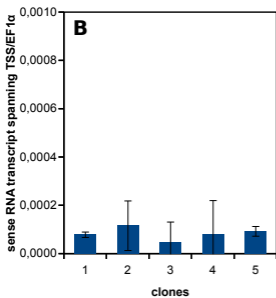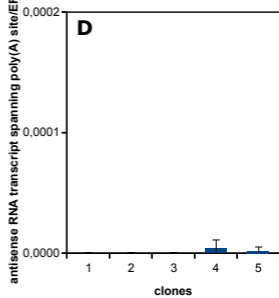

Supplement: Supplementary file 4 — Transcription of GFP gene in leaves of selected Arabidopsis thaliana transformants with pER8-GFP. RT-qPCR analysis of transcript levels in five selected lines untreated with β-estradiol. (A) The level of the sense GFP transcript; (B) the level of the sense transcript containing the region 50 nt upstream of transcription start site (TSS) of the inducible promoter; (C) the level of the antisense GFP transcript; (D) the level of the antisense transcript containing the region 50 nt downstream of the last poly(A) signal of T3A terminator. (PDF 39 kb) [file 12870_2018_1482_MOESM4_ESM.pdf]

1

2

3

4

5

(A) *Actin*

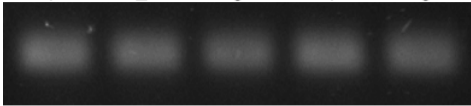

(B) *GFP*

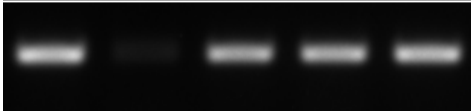

(C) noRT

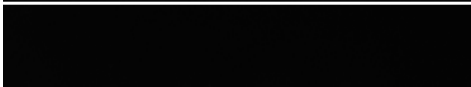

Supplement: Supplementary file 5 — Detection on polyA-tailed GFP transcripts in selected BY-2 calli transformed with pER8-GFP. Semiquantitative RT-PCR analysis of transcript levels in five independent calli untreated with β-estradiol (the same calli as in Fig. 5). cDNA was prepared using oligo dT primers. (A) The level of the Actin transcript (internal standard); (B) the level of the GFP transcript; (C) amplification of RNA samples that were not treated with reverse transcriptase to ensure that there was no DNA contamination. (PDF 41 kb) [file 12870_2018_1482_MOESM5_ESM.pdf]
